# Partnership Between Local Health Departments and Schools of Public Health or Public Health Programs: An Analysis of National Profiles of Local Health Departments

**DOI:** 10.3390/healthcare14070846

**Published:** 2026-03-26

**Authors:** Gulzar H. Shah, Katerina Massengale, Tran Ha Nguyen

**Affiliations:** 1 Jiann-Ping Hsu College of Public Health, Georgia Southern University, Statesboro, GA 30460, USA; gshah@georgiasouthern.edu; 2 School of Public Health, Augusta University, Augusta, GA 30912, USA; z123kat@gmail.com

**Keywords:** local health department, public health education, academic health department, public health education partnership

## Abstract

**Purpose:** This study examines (1) the change in partnership between local health departments (LHDs) and schools of public health or public health programs (SPHs/PHPs) from 2016 to 2019, and (2) the LHD characteristics associated with this partnership. **Background:** The Council on Education for Public Health updated accreditation criteria in 2016, shifting from core curricula to competencies to better prepare public health graduates for the workforce. Strong partnerships between LHDs and SPHs/PHPs can enhance practical training and employment opportunities for students, ultimately bolstering the public health workforce. **Methods:** We analyzed the 2016 and 2019 National Profiles of Local Health Departments, using descriptive statistics to evaluate partnership levels and multivariable logistic regression to identify LHD characteristics associated with collaboration. **Results:** The partnership between LHDs and SPHs/PHPs was suboptimal and unevenly distributed. Engagement in activities like formal training agreements and advisory roles declined. Notably, the presence of formal written agreements for staff training and active recruitment of SPH/PHP graduates by LHDs showed significant improvements (χ^2^ = 3.84; *p* = 0.049; χ^2^ = 8.19; *p* = 0.004). Factors such as top executive characteristics, workforce capacity, and governance context influenced these partnerships. **Conclusions:** The study identifies gaps in LHD engagement with SPHs/PHPs and highlights opportunities for advocacy. Addressing these gaps can lead to a more competent workforce, thereby benefiting both LHDs and SPHs/PHPs in their service to communities.

## 1. Introduction

The partnership between local health departments and academic institutions gained momentum in the 2000s [1,2], following the Institute of Medicine (IOM) report “The future of public health” in 1988 [3]. The report emphasized the gap between public health education in academia and the actual practice environments. The concept of these partnerships has evolved, leading to the formulation of “Academic Health Departments” in the 2010s [4]. This led to the development of “Academic Health Departments” (AHDs) in the 2010s, aiming to integrate academic learning with real-world public health needs.

Schools of public health (SPHs) and public health programs (PHPs) have long been engaging in research, training, and other partnerships with local health departments (LHDs) [5,6]. The introduction of the Public Health Accreditation Board (PHAB) in 2011 formalized the requirement for LHDs to partner with academic institutions to enhance public health capacity and accountability [7,8]. Research indicates that these partnerships are vital for advancing evidence-based practices and policies, benefiting both LHDs and SPHs/PHPs by improving research quality and addressing public health challenges [2,9,10,11,12,13].

Nonetheless, the nature of these relationships varies significantly across geographic areas, population levels, and experience levels, and they may be formal or informal. Some partnerships are highly formalized, often emerging through AHD models that institutionalize collaboration through shared governance, joint training, and coordinated research initiatives. These structured relationships tend to occur in regions with strong academic infrastructure and are supported by national efforts to strengthen academic–practice linkages [2]. In many areas, however, collaboration is semi-formal or informal, driven by specific projects, grant opportunities, or periodic needs such as community health assessments. Mid-sized and rural LHDs often rely on episodic partnerships due to limited staffing, geographic distance from universities, and fewer resources to sustain long-term engagement. These contextual differences contribute to significant variability in the depth and consistency of LHD–SPH relationships across the United States [14].

Despite their potential benefits, these partnerships face well-documented barriers. Barriers to effective collaboration include differing priorities, limited time and financial resources, and limited access for students, as well as factors such as proximity and organizational capacity [2,15]. Political turnover, funding instability, and cultural differences between academic and practice settings further complicate collaboration. These barriers are consistently identified across studies examining academic–practice partnerships and highlight the need for intentional strategies to strengthen and sustain these relationships [14].

When effectively structured, LHD–SPH collaborations enhance workforce development, support evidence-based practice, and improve the translation of research into community health action. Strengthening these partnerships is therefore essential for advancing public health capacity and improving population health outcomes [16].

In 2016, the Council on Education for Public Health (CEPH), an accreditation agency for schools of public health and public health programs, updated its accreditation criteria to emphasize competencies and the need for practical, hands-on experience for graduating students [17]. These competencies encompass the domains of evidence-based approaches to public health, healthcare systems, planning, and management to promote health, public health policy, leadership, communication, interprofessional and/or intersectoral practice, and systems thinking [11]. This shift encourages SPHs/PHPs to pursue formal partnerships with LHDs, yielding mutual benefits, including workforce preparedness and access to academic resources [18].

The change in CEPH criteria also established new expectations for applied learning, community engagement, and practice-oriented skill development, thereby creating structural incentives for SPHs/PHPs to strengthen partnerships with LHDs. Because LHDs serve as primary sites for experiential learning, field placements, and real-world data application, the revised criteria effectively increased the demand for formalized academic–practice collaboration. As a result, the CEPH 2016 standards function as a system-level mechanism that encourages SPHs/PHPs to expand practice linkages, develop joint training initiatives, and engage more deeply with public health agencies to meet accreditation expectations. These dynamics suggest that accreditation policy can shape the intensity, structure, and strategic orientation of LHD–SPH/PHP partnerships, positioning academic–practice collaboration as a critical component of workforce development and public health system strengthening.

The partnership between local health departments (LHDs) and schools or programs of public health (SPHs/PHPs) remains foundational for cultivating a competent public health workforce and preparing future leaders to address complex global health challenges [19]. The 2016 revision of the Council on Education for Public Health (CEPH) accreditation criteria did not function as a discrete causal intervention. Instead, it introduced a structural expectation for competency-based education that operates as a contextual pressure within the broader public health system. This normative shift created an external impetus for academic institutions and health departments to reassess partnership strategies in response to evolving accreditation standards. Within this context, the present study examines how systemic pressures align with patterns of engagement between LHDs and SPHs/PHPs. It also identifies organizational characteristics that may mediate responsiveness to accreditation-driven dynamics, including jurisdictional size, governance structure, leadership expertise, and workforce capacity.

Changes in LHD–SPH/PHP partnerships from 2016 to 2019 are influenced by accreditation pressures. SPHs/PHPs are directly subject to CEPH criteria and undergo formal accreditation cycles and external reviews, which drive them to enhance practice-based training and engage with public health agencies. This urgency leads to more rapid adjustments, particularly as accreditation deadlines approach. Conversely, LHDs are indirectly affected by these pressures, as they are not governed by CEPH standards and face no formal consequences for failing to expand partnerships. Their response is influenced by internal factors like staffing and operational demands. Consequently, changes within LHDs may occur more slowly and unevenly, even if SPHs/PHPs adapt rapidly.

This asymmetry helps explain why some partnership effects may be detectable within the 2016–2019 period—driven largely by academic institutions—while broader, system-level changes may not yet be fully observable. The differential pressures placed on SPHs/PHPs versus LHDs highlight the importance of considering organizational context when assessing the early impacts of accreditation policy on academic–practice collaboration.

By illuminating these structural and contextual influences, the study offers a meaningful policy contribution: it highlights opportunities for policymakers to formalize academic–practice partnerships, invest in sustainable workforce development pipelines, and reduce capacity disparities across jurisdictions. Strengthening these partnerships has the potential to enhance evidence-based decision-making, expand the public health workforce, and reinforce the nation’s public health infrastructure through more intentional and equitable collaboration.

## 2. Materials and Methods

### 2.1. Data Source

This research utilized the 2016 and 2019 National Profile of Local Health Departments Surveys. The National Association of County and City Health Officials (NACCHO) is a non-profit organization that represents local health departments across the United States (US). Since 1989, NACCHO has been conducting the National Profile of Local Health Departments, commonly referred to as the “Profile Study”, every three years. The purpose of the Profile Study is to create a comprehensive and accurate description of LHDs’ infrastructure and practices. To identify the LHDs included in the profile studies, NACCHO uses a database derived from previous ones, along with consultations with state health agencies and local health officials’ associations. All LHDs in the study population received core questionnaires, while selected groups of LHDs received one of two sets of supplementary questions, referred to as modules. NACCHO employs stratified random sampling without replacement, with strata defined by the population size served by each LHD. We refer to the study website for additional information on NACCHO profile studies [20]. The Profile Study employs a standardized survey instrument that has been refined through multiple cycles of expert consultation, cognitive testing, and pilot assessments. These iterative methodological improvements support the content validity and reliability of the measures used to assess LHD infrastructure and activities.

The 2016 Profile Study included 2533 local health departments (LHDs), of which 1952 (76%) completed the survey. In contrast, the 2019 Profile Study included 2453 LHDs, of which 1496 (61%) completed the survey. The survey items analyzed in this research were included in Module 1, which received 484 responses in 2016 and 380 in 2019.

### 2.2. Measures

To measure LHDs’ engagement with SPHs/PHPs, we used specific survey items included in both the 2016 and 2019 surveys. The dependent variable came from the 2019 Profile Study. It was measured using a 9-item scale of collaboration activities (coded 0 or 1), yielding a count variable ranging from 0 to 9. The activities include the following: (1) agreements or policies providing LHDs with access to scientific and professional journals; (2) consulting roles by SPH/PHP faculty or staff; and (3) acceptance of students as trainees, interns, or volunteers. Additional activities include (4) graduate recruitment, (5) formal training agreements for LHD staff, (6) LHD staff serving as faculty, (7) advisory roles, (8) collaborative research, and (9) contracts for public health service provision. The nine-item composite index was created to measure the range of collaboration between LHDs and SPH/PHPs. This summary index is intended to reflect the scope of collaborative activities rather than their depth or resource intensity. All activities were weighted equally. They may have differed in institutional cost, complexity, and strategic value. Therefore, this measure indicates the diversity of engagement instead of the intensity of partnerships.

Based on the existing literature [21], independent variables representing the characteristics of Local Health Departments (LHDs) were organized into five blocks: (1) LHD top executive characteristics, (2) workforce capacity, (3) community health planning and accreditation, (4) partnerships and cross-jurisdictional sharing, and (5) governance context. Additionally, two control variables were defined based on the LHD’s population size served (small, medium, or large) and governance type (state, local, or shared).

The block on LHD top executive characteristics included their work status (part-time or full-time), prior experience (yes or no), race (White, Black, American Indian and Alaska Native, Asian, or other), gender (male or female), age (in years), the highest degree held (associate, bachelor, master, or doctorate), and the specialty of the degree (medical, nursing, public health, or other).

Workforce capacity was assessed through the number of full-time equivalent (FTE) positions filled, the number of FTE epidemiologists employed, and the number of FTE vacancies.

The community health planning and accreditation block consisted of whether the LHD completed a community health assessment (yes or no), participated in a community health improvement plan (yes or no), has an agency-wide strategic plan (yes or no), and its application status with the Public Health Accreditation Board (not applying, accredited, submitted application, registered to pursue accreditation, or planning to apply for accreditation).

The partnerships and cross-jurisdictional sharing block included four scaled variables and one dichotomous variable. The scaled variables, from 0 to 22, represented the extent of the LHD’s involvement with local organizations in sharing personnel and resources, having written agreements, scheduling regular meetings, and exchanging information. The dichotomous variable indicated whether the LHD shared resources—such as funding, staff, or equipment—with one or more other LHDs on a continuous, recurring, non-emergency basis.

The governance context block involved the presence of the Local Board of Health (LBOH) (yes or no), the scope of their authority (governing or advisory), the extent of their involvement in the National Association of Local Boards of Health (NALBOH) Six Governance Functions (on a scale of 0 to 6), and the activities for which the board holds final authority (on a scale of 0 to 10). Details of variables used to address research question 2 in this study are provided in Appendix A.

### 2.3. Data Analysis

Descriptive statistics were computed as appropriate. The z-test for proportions was used to compare the proportions of “yes” responses in the 2016 and 2019 surveys to determine whether there were significant differences between the two periods, answering Research Question 1.

For Research Question 2, due to the over-dispersed Poisson distribution of the dependent variable (mean = 1.83; variance = 4.29) and a high proportion of zeros (37%), an inflated negative binomial regression—a variant of Poisson regression—was conducted to identify factors associated with LHDs’ engagement with SPH/PHP with each independent variable block with a total of five regression models. All analyses were performed using STATA version 19 (STATA Corp, College Station, TX, USA), with a significance threshold of 0.05 (*p* < 0.05).

We used the post-stratification weights developed by NACCHO scientists in our analyses. These survey weights are designed to account for variation in response patterns by jurisdiction size, governance type, and geographic region, ensuring that each responding LHD accurately reflects its appropriate share of the national population of health departments. Since these weights are applied at the organizational (case) level rather than at the individual variable level, each variable contributes equally to the analysis once the NACCHO-provided weight is included. This method aligns with NACCHO’s original methodological design and avoids introducing additional weighting schemes that could compromise national representativeness [22].

## 3. Results

**Figure 1:**
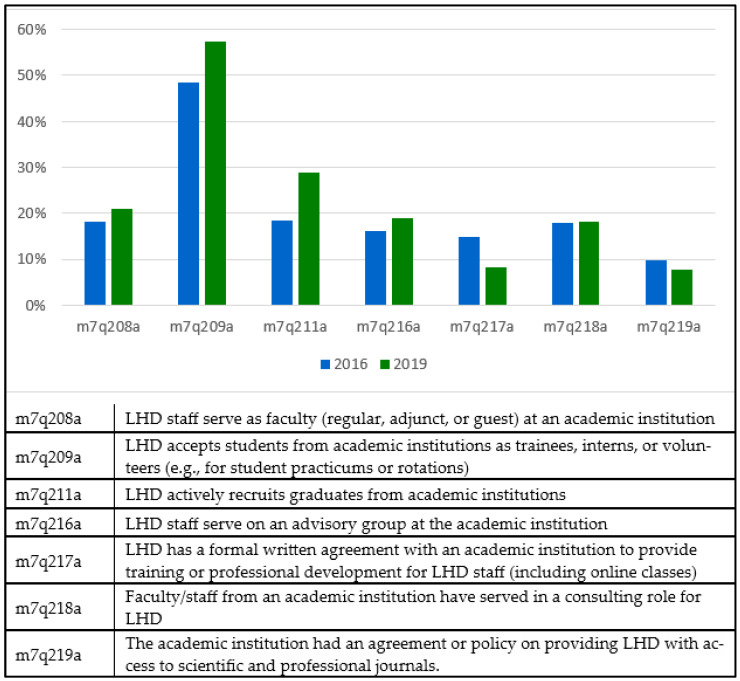
At LHD Engagement in Activities with schools or programs of public health (SPHs/PHPs) in 2016 vs. 2019.

Figure 1 illustrates the percentage of local health departments (LHDs) involved in activities with schools of public health (SPHs) and public health programs (PHPs), based on data from the 2016 and 2019 surveys. Our results show that between 2016 and 2019, the involvement of LHDs with SPH/PHPs varied. Accepting students as trainees, interns, or volunteers was the most common activity, which increased from 48% in 2016 to 57% in 2019. The least common activity, in contrast, was providing LHDs access to scientific journals, which declined slightly from 10% to 8%. Overall, collaboration was suboptimal but unevenly distributed, as indicated by the modest and declining engagement in activities such as formal training agreements, faculty exchanges, and advisory roles.

Table 1 presents the results of the z-test for proportions, comparing the engagement levels of LHDs with those of SPHs and PHPs to determine whether there is a significant difference between the two surveys. Among the seven activities examined, LHD has a formal written agreement with SPH/PHP to provide training or professional development for LHD staff (including online classes) and LHD actively recruits graduates from SPH/PHP, showing significant changes between the 2016 and 2019 surveys (χ^2^ = 3.84; *p* = 0.049; χ^2^ = 8.19; *p* = 0.004, respectively). While LHDs increased recruitment from SPHs/PHPs, they reduced the number of formal written agreements for staff training or professional development.

**Figure 2:**
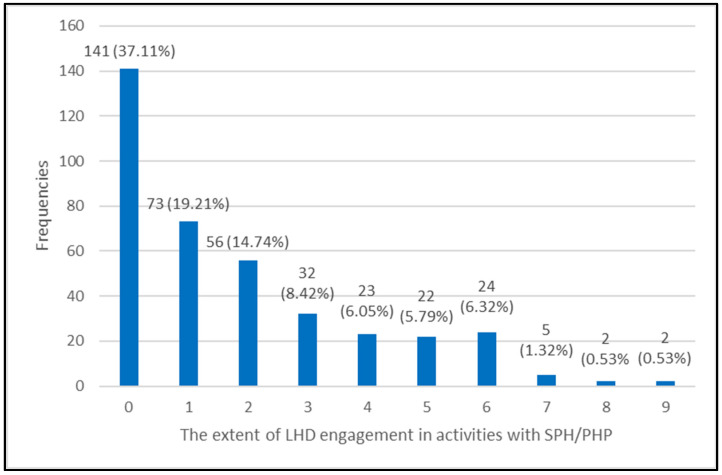
Number of collaboration activities among LHDs and schools or programs of public health (SPHs/PHPs), in the 12 months preceding the 2019 Profile Study.

Figure 2 presents the number of collaboration activities among LHDs and SPH/PHP, which follows a Poisson distribution. In the 12 months preceding the 2019 study, 141 (37%) of LHDs did not engage in any activities with SPH/PHP. On a scale of 0 to 9, the mean of activities reported by participating LHDs was 1.83, with a median of 1.

Table 2 shows the descriptive statistics for the variables used in the zero-inflated negative binomial regression models. Meanwhile, Table 3 displays the results of these regressions, which explore the factors influencing the levels of engagement of LHDs in activities with SPH/PHP. The interpretation of the zero-inflated negative binomial regression relies on the estimation of incidence rate ratios (IRR). These ratios represent the proportional increase or decrease in the dependent variable associated with each independent variable. The actual change is determined by the magnitude of the IRR, which will be either above or below 1.

Table 3 presents the results of these regressions, which examine the factors influencing LHD engagement in activities with SPH/PHP. The interpretation of the zero-inflated negative binomial regression relies on the estimation of incidence rate ratios (IRR). These ratios represent the proportional increase or decrease in the dependent variable associated with each independent variable. The actual change is determined by the magnitude of the IRR, which will be either above or below 1.

Among the characteristics of LHDs’ leaders, the highest level of education attained showed a strong positive association with LHDs’ engagement in SPH/PHP activities, with higher terminal degrees corresponding to greater engagement. LHDs led by individuals with master’s and doctoral degrees had engagement odds that were 2.214 and 2.874 times higher, respectively, than those of LHDs led by individuals with associate degrees.

The analysis of the relationship between workload capacity factors and LHD’s engagement with SPH/PHP activities revealed very weak positive and negative associations. The current number of vacancies was not statistically significant. The number of FTEs currently filled showed a weak association with an IRR of 1.001; for each one-unit increase in FTEs employed, the incidence rate increases by 0.1%. Conversely, the number of FTE epidemiologists employed showed a weak negative association (IRR = 0.984). For each one-unit increase in this continuous variable, the engagement rate with SPH/PHP activities decreases by 1.6 percentage points.

While LHDs’ community health planning did not yield a statistically significant association with their engagement with SPH/PHP activities, their PHAB accreditation status demonstrates positive associations. LHDs accredited by PHAB and those planning to apply for accreditation exhibited engagement odds 1.853 and 1.969 times higher, respectively, than those of LHDs not applying for PHAB accreditation.

LHDs’ partnerships with local organizations were also positively associated with their engagement with SPH/PHP activities. LHDs that have written agreements with community partners and those that regularly meet with them exhibit higher engagement rates. For every local organization with which LHD has entered into an agreement, there is a 1.048 increase in engagement rate, equivalent to a 4.8% increase. With one additional local organization with which LHD regularly has meetings, there is an increase in engagement rate of 1.028, equivalent to a 2.8% increase.

In the context of LHD governance, negative associations with LHDs’ engagement with SPH/PHP activities were revealed. The presence of LBOH lowered LHDs’ engagement rate by 0.441 times or 55.9% in activities with SPH/PHP. In addition, with every LHD activity, LBOH had the final authority; the engagement rate with SPH/PHP activities decreased by 7.3% according to an IRR of 0.927.

For each of the five zero-inflated negative binomial regressions, LHDs’ governance type and their population size served as control variables. LHDs’ engagement odds in activities with SPH/PHP consistently increase with the size of the population served. LHDs serving medium and large populations show strong positive associations with engagement in SPH/PHP activities compared with those serving small populations, with engagement odds up to 2.628 times higher for medium-sized populations and 4.786 times higher for larger populations. Regarding governance types, LHDs with local or shared governance are positively associated with higher engagement rates in activities with SPH/PHP compared with LHDs with state governance, with engagement rates up to 1.700 and 2.067 times higher, respectively.

## 4. Discussion

This study examined the partnership (or lack thereof) between local health departments (LHDs) and schools of public health/public health programs (SPHs/PHPs) following the 2016 revision to the Council on Education for Public Health (CEPH) accreditation criteria for public health education. The study also explored the characteristics of LHDs that are associated with their engagement with SPHs/PHPs. Although engagement levels were suboptimal, showing a modest increase in most partnership areas examined during 2016 and 2019, this trend should not be interpreted as evidence of the ineffectiveness of the CEPH 2016 criteria reform. Instead, the observed decline may reflect the limited time available for systemic adaptation and organizational realignment in response to structural pressures introduced by the accreditation changes. Transformative shifts in educational standards and interorganizational partnerships often require extended periods for implementation, resource allocation, and cultural integration. Consequently, short-term fluctuations in engagement likely represent transitional dynamics rather than a failure of the reform itself.

Another important finding concerns workforce composition. We found that our IRR was small (IRR = 1.001), suggesting a weak association between the number of FTE positions filled and engagement. However, in context, one FTE is a small staffing unit, and many Local Health Departments (LHDs) employ thousands of FTEs. Thus, even a small change in the number of positions filled can reflect a significant increase in workforce capacity, and the effect size may appear small but still affect departments’ ability to sustain or expand academic partnerships. In addition, we found a negative association between the number of epidemiologists and partnership engagement. This may reflect the operational demands placed on epidemiologists, who are often deeply engaged in surveillance, reporting, and preparedness activities. That may leave limited capacity for external collaborations. This pattern may also relate to department size or structure, in which epidemiologists’ roles are primarily internal.

Our study shows that engagement in partnerships is associated with governance structures. The LBOHs may play a critical role in restricting the strategic and administrative flexibility of LHDs. For instance, the boards with greater authority are likely to introduce additional oversight, procedural review, or legal requirements for external collaborations. While LBOH governance functions enhance accountability and transparency, they can also limit LHDs’ decision-making. Research shows that variation in LBOH governance functions affects how local agencies balance compliance with innovation [22,23]. LHDs operating under more restrictive governance may therefore have fewer opportunities to create and maintain discretionary partnerships with academic institutions.

Public health literature has thoroughly documented the ongoing challenges faced by academic health departments (AHDs). These challenges include logistical barriers, funding issues, and competing priorities that often shift over time [23]. According to the NACCHO Forces of Change Study 2018, which evaluates changes in the capacity and activities of LHDs driven by public health trends, one-third of all LHDs lost at least one staff position due to layoffs or attrition in 2017. One in five LHDs (21%) reported a decrease in their budget compared to the previous fiscal year [24]. The cumulative adverse effects of job losses and funding cuts can hinder the partnerships between LHDs and SPHs/PHPs. Moreover, the varying long-term goals of students, the constraints stemming from diverse cultures within academic institutions and public health agencies, the challenge of creating mutually beneficial learning opportunities, and shifts in organizational priorities all contribute to the difficulties in fostering partnerships within academic health departments [23].

Local Health Departments (LHDs) that serve large populations are more likely to collaborate with SPHs/PHPs. In urban or high-population areas, LHDs have better access to these institutions, funding for joint initiatives, and specialized staff and infrastructure to support collaboration. As a result, these departments are often designated as Academic Health Departments (AHDs), which are formal partnerships aimed at enhancing training, research, and workforce development [25,26].

Our study revealed that LHDs governed by local or shared authority (between local and state) engaged more with SPH/PHP than those governed solely by state authorities. In this regard, public health literature offers inconclusive evidence on whether LHDs governed by local authorities interact more with academic institutions than those governed by state authorities [16,27]. Even though partnerships are established at both levels for various reasons, LHDs governed by local authorities often serve as key points of practice-based interaction with academic units. In contrast, those governed by state authorities may use academia primarily for research and policy purposes. More research is necessary to clarify this area.

Several limitations should be considered when interpreting the results. This study analyzes the partnership between LHDs and SPHs/PHPs before and after the introduction of the 2016 CEPH criteria, using data from the 2016 and 2019 National Profile of LHD Studies. First, differences in response rates across survey years may bias the comparison. Response rates declined from 76% in 2016 to 61% in 2019 and were lower among small LHDs than among larger departments, particularly those serving populations of over 500,000 [28,29,30]. Secondly, the study relies on a cross-sectional secondary dataset; therefore, it inherits the limitations of the National Profile Studies, including the inability to establish causation. Thirdly, NACCHO Profile studies reflect the perspective of LHDs. We were unable to verify how SPHs/PHPs view these collaborations. Fourth, since the introduction of the CEPH criteria in 2016, three years may not be sufficient for quantifiable impacts on partnerships to manifest.

The Module questions are administered to a stratified random sample designed to be nationally representative, although lower response rates may limit the extent to which the findings can be fully generalized. Although NACCHO’s case-level weights help adjust for response patterns, some nonresponse bias may remain. Therefore, the generalizability of our findings is strongest at the national level but may be more limited for specific subgroups. Sensitivity checks were not performed, as NACCHO provides the validated post-stratification weights for all analyses; any additional weighting or subsampling adjustments would be unlikely to affect the representativeness of this dataset.

Although this study has these limitations, it provides national data with high generalizability and offers new insights into the partnership between LHDs and SPHs/PHPs. It is important to note that the data were collected prior to the onset of the COVID-19 pandemic, which profoundly reshaped the political and operational dynamics of the public health landscape. The pandemic introduced unprecedented challenges and priorities, influencing resource allocation, governance structures, and interorganizational collaboration in ways that could not have been anticipated during the study period. Conducting further research in this area would provide updated insights into the partnership’s evolution under these altered conditions. Moreover, the pandemic accelerated the adoption of virtual modalities such as video conferencing, telehealth, and remote engagement. Such adoption highlighted the need to examine how these technological and systemic shifts have redefined the relationship between LHDs and SPHs/PHPs.

While this manuscript was being developed, a significant shift in U.S. politics would have a profound impact on the public health community. Under the Trump Administration, the Centers for Disease Control and Prevention (CDC) began to retract $11.4 billion in COVID-era grants in early 2025, affecting nearly all aspects of public health operations [31,32]. Although COVID-19 led to a temporary increase in funding, the aftermath has left many LHDs struggling to maintain essential services and with limited capacity to respond to future crises [33]. The effects of this public health challenge on the partnership between LHDs and SPHs/PHPs warrant further research.

### Implications for Practice

This study underscores the suboptimal relationship between Local Health Departments (LHDs) and Schools of Public Health and Public Health Programs (SPH/PHP), which appeared to weaken from 2016 to 2019, with notable declines recorded across nearly all categories of involvement during this period. These findings should not be interpreted as evidence of the ineffectiveness of the CEPH 2016 criteria reform. Rather, the observed trend likely reflects the limited time available for organizations to adapt to the structural pressures introduced by the accreditation changes. Transformative shifts in educational standards and interorganizational partnerships typically require extended periods for implementation, resource allocation, and cultural integration. Consequently, short-term declines in engagement may reflect temporary adjustment processes rather than a failure of the reform. Proactive measures remain crucial for strengthening partnerships between SPHs/PHPs and LHDs. Opportunities exist for SPHs/PHPs to advocate for and forge collaborations within their regions, ensuring compliance with CEPH’s competency-based criteria while enhancing community service, creating networking opportunities for students, and cultivating a skilled public health workforce. Engagement with state agencies can further support these efforts, fostering a dynamic, responsive workforce equipped to address current challenges and prepare for future opportunities.

## 5. Conclusions

This study provides evidence of persistent gaps in partnerships between Local Health Departments (LHDs) and Schools or Programs of Public Health (SPH/PHP). Overall engagement remains limited, uneven across jurisdictions, and declined in some partnership domains between 2016 and 2019, with only modest improvements in activities such as student placements and graduate recruitment. These patterns likely reflect temporary organizational shifts associated with the structural pressures introduced by accreditation changes, rather than a lack of commitment or capacity to adapt.

Factors such as leadership education level, accreditation status, and the size of the population served were positively associated with stronger partnerships, whereas governance type was associated with notable disparities. Although certain forms of engagement—such as graduate recruitment—have increased, other formal mechanisms, including training agreements, have declined. These findings underscore the need for targeted strategies and incentives to strengthen institutional collaborations, particularly for LHDs serving smaller jurisdictions and those under state governance. In an era of constrained budgets and heightened accountability driven by PHAB accreditation, fostering robust academic–practice partnerships remains pivotal to advancing the nation’s public health infrastructure and workforce readiness. Future research should employ longitudinal designs to capture the effects of the CEPH reform, as short-term trends may not fully reflect the long-term impact of structural changes on partnership dynamics.

## Figures and Tables

**Table 1:** Outcomes of the Z-test for proportions, comparing the engagement levels of LHDs with SPHs/PHPs in 2016 versus 2019.

Variable	Attribute	Z-Score, Profile Study 2016	Z-Score, Profile Study 2019	χ^2^ and *p*
SPH/PHP has an agreement or policy on providing LHD with access to scientific and professional journals	Yes	(47) 10%	(29) 8%	χ^2^ = 0.44 *p* = 0.505
Faculty/staff from SPH/PHP have served in a consulting role for LHD	Yes	(86) 18%	(68) 18%	χ^2^ = 0.00 *p* = 1.000
LHD has a formal written agreement with SPH/PHP to provide training or professional development for LHD staff (including online classes)	Yes	(72) 15%	(31) 8%	χ^2^ = 3.84 ***p* = 0.049**
LHD staff serve in an advisory group at SPH/PHP	Yes	(78) 16%	(71) 19%	χ^2^ = 0.67 *p* = 0.413
LHD actively recruits graduates from SPH/PHP	Yes	(89) 18%	(108) 29%	χ2 = 8.19 ***p* = 0.004**
LHD accepts students from SPH/PHP as trainees, interns, or volunteers (e.g., for students’ practicums or rotations)	Yes	(234) 48%	(214) 57%	χ^2^ = 3.24 *p* = 0.072
LHD staff serve as faculty (regular, adjunct, or guest) at SPH/PHP	Yes	(88) 18%	(78) 21%	χ^2^ = 0.61 *p* = 0.435

Abbreviations: LHD, Local health department; SPH, schools of public health; PHP, public health program; χ^2^, chi-square; *p*, *p*-value. Note: **Boldface** indicates statistical significance of estimates at *p*-value < 0.05.

**Table 2:** Descriptive statistics of the study variables used in the zero-inflated negative binomial (N = 380). Profile Study 2019.

Variables	N or Mean	% or SD
Dependent Variable		
The Extent of LHD engagement in activities with SPHs/PHPs (scale from 0 to 9)	1.83	2.07
Independent Variables		
Model 1 (Top Executive Characteristics)		
Work status Part time Full time	17 357	4.55 95.45
Prior experience as the top executive of an LHD? No Yes	73 293	19.95 80.05
Race White Black AIAN Asian Other	321 33 2 13 4	86.06 8.85 0.54 3.49 1.07
Gender Male Female	136 238	36.36 63.64
Age (in years, ranging from 29 to 83)	52.22	9.84
Highest degree held Associate Bachelor Master Doctorate	20 99 185 59	5.51 27.27 50.96 16.25
Specialty Medical Nursing Public Health Other	31 74 110 158	8.31 19.84 29.49 42.36
Model 2 (Workforce Capacity)		
Number of FTE currently filled (ranging from 0 to 5,592)	108	390
Number of current FTE vacancies (ranging from 0 to 798)	13	65
Number of FTE epidemiologists employed (ranging from 0 to 391)	3	24
Model 3 (Community Health Planning and Accreditation)		
Completion of the community health assessment No Yes	46 334	12.11 87.89
Participation in the community health improvement plan No Yes	80 300	21.05 78.95
Having an agency-wide strategic plan No Yes	94 284	24.74 75.26
PHAB status Not applying Accredited Submitted application Registered to pursue accreditation Plan to apply for accreditation	222 87 24 13 34	58.42 22.89 6.32 3.42 8.95
Model 4 (Partnerships and Cross-jurisdictional Sharing)		
Shared resources with community partners (scale from 0 to 22)	4.02	4.92
Had written an agreement with community partners (scale from 0 to 22)	3.49	3.86
Regularly met with community partners (scale from 0 to 22)	6.93	5.31
Exchanged information with community partners (scale from 0 to 22)	13.36	6.25
Share resources with other LHD(s) No Yes	177 203	46.58 53.42
Model 5 (Governance Context)		
Presence of LBOH No Yes	106 271	28.12 71.88
LBOH authority Governing Advisory	202 69	74.54 25.46
Involvement in NALBOH’s 6 Functions of Governance (scale from 0 to 6)	2.59	2.20
LBOH’s final authority on LHDs’ activity (scale from 0 to 6)	6.87	2.82
Control variables		
LHD Governance Type State Local Shared	64 273 43	16.84 71.84 11.32
Size of population served Small (<50,000) Medium (50,000–499,999) Large (≥500,000)	170 155 55	44.74 40.79 14.47

Abbreviations: LHD, Local Health Department; SPH, School of Public Health; PHP, Public Health program; PHAB, Public Health Accreditation Board; LBOH, Local Board of Health; NALBOH, National Association of Local Boards of Health; N, Total response; n, number of responses to each item; %, proportion; SD, Standard Deviation; AIAN, American Indian or Alaska Native; FTE, Full-Time Equivalent.

**Table 3:** Zero-inflated Negative Binomial Regression Model for predicting Factors associated with the number of LHDs’ collaboration activities with SPH/PHP, 2019.

LDH Characteristics	IRR	*p*-Value	(95% CI)
Model 1 (Top Executive Characteristics)			
Work status Part time Full time	-- 1.836	-- 0.235	-- (0.890, 3.788)
Prior experience as the top executive of an LHD? No Yes	-- 0.889	-- 0.598	-- (0.693, 1.140)
Race White Black AIAN Asian Other	-- 1.085 0.967 0.585 2.495	-- 0.839 0.934 0.084 0.304	-- (0.783, 1.502) (0.107, 8.753) (0.342, 0.998) (0.933, 6.667)
Gender Male Female	-- 0.878	-- 0.474	-- (0.706, 1.093)
Age (in years)	1.004	0.310	(0.993, 1.015)
Highest degree held Associate Bachelor Master Doctorate	-- 0.855 **2.214** **2.874**	-- 0.299 0.034 0.002	-- (0.384–1.906) (1.012, 4.841) (1.248, 6.614)
Specialty Medical Nursing Public Health Other	-- 0.689 1.022 0.980	-- 0.868 0.153 0.088	-- (0.424, 1.118) (0.694, 1.504) (0.643, 1.494)
Model 2 (Workforce Capacity)			
Number of FTE currently filled	**1.001**	0.021	(1.000, 1.002)
Number of current FTE vacancies	0.995	0.061	(0.995, 1.002)
Number of FTE epidemiologists employed	**0.984**	0.041	(0.972, 0.996)
Model 3 (Community Health Planning and Accreditation)			
Completion of the community health assessment No Yes	-- 1.114	-- 0.707	-- (0.695, 1.787)
Participation in the community health improvement plan No Yes	-- 1.366	-- 0.131	-- (0.934, 1.996)
Having an agency-wide strategic plan No Yes	-- 1.209	-- 0.729	-- 0.897, 1.631)
PHAB status Not applying Accredited Submitted application Registered to pursue accreditation Plan to apply for accreditation	-- **1.853** 1.413 1.225 **1.969**	-- 0.001 0.299 0.304 0.002	-- (1.454, 2.362) (0.953, 2.097) (0.751, 1.997) (1.434, 2.704)
Model 4 (Partnerships and Cross-jurisdictional Sharing)			
Shared resources with community partners (scale)	1.020	0.396	(0.999, 1.042)
Had written an agreement with community partners (scale)	**1.048**	0.011	(1.020, 1.076)
Regularly met with community partners (scale)	**1.028**	0.014	(1.005, 1.052)
Exchanged information with community partners (scale)	1.015	0.209	(0.996, 1.036)
Share resources with other LHD(s) No Yes	-- 1.091	-- 0.374	-- (0.902, 1.320)
Model 5 (Governance Context)			
Presence of LBOH No Yes	-- **0.441**	-- 0.049	-- (0.237, 0.820)
LBOH authority Governing Advisory	-- 1.295	-- 0.341	-- (0.888, 1.887)
Involvement in NALBOH’s 6 Functions of Governance (scale)	1.067	0.074	(0.988, 1.153)
LBOH’s final authority on LHDs’ activity (scale)	**0.927**	0.027	(0.868, 0.990)
Control Variables			
LHD Governance Type State Local Shared	-- **1.700** **2.067**	-- 0.003 0.044	-- (1.217, 2.375) (1.359, 3.143)
Size of population served Small (<50,000) Medium (50,000–499,999) Large (≥500,000)	-- **2.628** **4.786**	-- <0.001 <0.001	-- (2.053, 3.364) (3.565, 6.425)

Note: -- indicate the reference categories. **Boldface** indicates statistical significance of estimates at *p*-value < 0.05. Abbreviations: LHD, Local Health Department; SPH/PHP, Schools of Public Health/Public Health Programs; PHAB, Public Health Accreditation Board; LBoH, Local Board of Health; NALBOH, National Association of Local Boards of Health; FTE, Full-Time Equivalent; IRR, Incidence Rate Ratio; CI, Confidence Interval.

## Data Availability

The data used in this study are publicly available through the Inter-university Consortium for Political and Social Research (ICPSR) repository. Researchers may access the datasets via https://www.icpsr.umich.edu/ (accessed on 13 June 2025).

## Appendix A. Variables’ Operationalization

**Table:**

Study Variables	Survey Item	Survey Choice	Study Variable’s Categories
**Dependent Variable**			
The Extent of LHD engagement in activities with SPHs/PHPs	Indicate whether your LHD has been engaged in the following activities in the past year with Schools or Programs of Public Health	1. SPH/PHP has an agreement or policy on providing LHD with access to scientific and professional journals. 2. Faculty/staff from SPH/PHP have served in a consulting role for LHD. 3. LHD accepts students from SPH/PHP as trainees, interns, or volunteers (e.g., for students’ practicums or rotations). 4. LHD actively recruits graduates from SPH/PHP. 5. LHD has a formal written agreement with SPH/PHP to provide training or professional development for LHD staff (including online classes). 6. LHD staff serve as faculty (regular, adjunct, or guest) at SPH/PHP. 7. LHD staff serve in an advisory group at SPH/PHP. 8. SPHs/PHPs instruction collaborates with LHDs on research studies. 9. LHD contracts with SPH/PHP to provide public health services.	Scale from zero to 9
**Independent Variables**			
*LHD top executive characteristics*			
Work Status	What is the work status for the top executive?	- Part-time - Full-time	- Part-time - Full-time
Prior experience	Is this their first position as the top executive of an LHD?	- No - Yes - Unknown (excluded)	- No - Yes
Race	What is the race of the person in the top executive position?	- White - Black or African American - AIAN - Asian - NH or other PI (no response) - Other	- White - Black or African American - AIAN - Asian - Other
Gender	What is the gender of the person in the top executive position?	- Male - Female - Other (no response) - Prefer not to answer (no response)	- Male - Female
Age	What is the age of the person in the top executive position?	Whole numbers	Whole number in years
Highest degree held	What is the highest degree your top executive holds?	- Associate - Bachelor - Master - Doctorate	- Associate - Bachelor - Master - Doctorate
Specialty	Does your top executive hold a degree in the following specialization?	- Medical - Nursing - Public health - None of the above	- Medical - Nursing - Public health - Other
*Workforce capacity*			
Number of FTE filled	Indicate the number of individuals and the number of FTE who currently work for your LHD. If your LHD has any vacancies, please indicate the number of FTE vacancies. (To include all regular full-time, part-time, contractual, and seasonal employees.)	Whole numbers	Whole numbers
Number of FTE vacancies		Whole numbers	Whole numbers
Number of FTE epidemiologists employed	Number of FTEs currently employed. (Epidemiologist/Statistician)	Whole numbers	Whole numbers
*Community health planning and accreditation*			
Completion of the community health assessment	Has a community health assessment been completed for your LHD’s jurisdiction?	(1) Yes, within the last three years (2) Yes, more than three years ago, but within the past five years (3) Yes, more than five years ago (4) No, but plan to complete one within the next year (5) No	- Yes (choices #1–3) - No (choices #4–5)
Participation in the community health improvement plan	Has your LHD participated in developing a health improvement plan for your community?	(1) Yes, within the last three years (2) Yes, more than three years ago, but within the past five years (3) Yes, more than five years ago (4) No, but plan to participate or develop one within the next year (5) No	- Yes (choices #1–3) - No (choices #4–5)
Having an agency-wide strategic plan	Has your LHD developed a comprehensive, agency-wide strategic plan?	(1) Yes, within the last three years (2) Yes, more than three years ago, but within the past five years (3) Yes, more than five years ago (4) No, but plan to develop a strategic plan within the next year (5) No	- Yes (choices #1–3) - No (choices #4–5)
PHAB status	Which of the following best describes your LHD’s participation in the PHAB’s national accreditation program for LHDs?	(1) My LHD has been accredited by PHAB (2) My LHD has submitted an application for PHAB accreditation (3) My LHD has registered in e-PHAB in order to pursue accreditation (4) My LHD plans to apply for PHAB accreditation, but has not yet registered in e-PHAB (5) My LHD has not decided whether to apply for PHAB accreditation (6) My LHD has decided not to apply for PHAB accreditation	- Not applying (choice #5–6) - Accredited - Submitted application - Registered to pursue accreditation - Plan to apply for accreditation
*Partnerships and cross-jurisdictional sharing*			
Shared resources with community partners	Check every way your LHD has worked with each organization in the past year. (Shared personnel and resources)	1. Business (community-based) 2. Colleges or universities 3. Community health centers 4. Community-based nonprofits 5. Cooperative extensions 6. Criminal justice system 7. Economic and community development agencies 8. Emergency responders 9. Faith communities 10. Health insurers 11. Hospitals 12. Housing agencies 13. K–12 schools 14. Libraries 15. Local planning agency 16. Media 17. Mental health/Substance abuse providers 18. Parks and recreation 19. Physician practices/Medical groups 20. Transportation 21. Tribal gov’t agencies 22. Veterinarians	Scale from zero to 22
Had written an agreement with community partners	Check every way your LHD has worked with each organization in the past year. (Written agreement)		Scale from zero to 22
Regularly met with community partners	Check every way your LHD has worked with each organization in the past year. (Regularly scheduled meetings)		Scale from zero to 22
Exchanged information with community partners	Check every way your LHD has worked with each organization in the past year. (Exchanged information)		Scale from zero to 22
Shared resources with other LHD(s)	Currently, does your LHD share resources (such as funding, staff, or equipment) with one or more other LHDs on a continuous, recurring, non-emergency basis?	- No - Yes	- No - Yes
*Governance context*			
Presence of a LBOH	Does your LHD have one or more local boards of health?	- No - Yes - Unknown (excluded)	- No - Yes
LBOH authority	Please choose what best describes your local board of health	- Advisory - Governing	- Advisory - Governing
Involvement in NALBOH’s six functions of governance	Which of the following of NALBOH’s Six Functions of Governance does your local board of health utilize on a continuous basis?	1. Continuous improvement 2. Legal authority 3. Oversight 4. Partner engagement 5. Policy development 6. Resource stewardship	Scale from zero to 6
LBOH’s final authority on LHD’s activities	Check each action that your local board of health has final authority to do.	1. Advise LHD or elected officials on policies, programs, and budgets 2. Approve the LHD budget 3. Adopt public health regulations 4. Hire or fire agency head (e.g., top executive, medical director/health officer 5. Impose or enforce quarantine or isolation orders 6. Impose taxes for public health 7. Request a public health levy 8. Set policies, goals, and priorities that guide the LHD 9. Set and impose fees 10. Other	Scale from zero to 10
**Control variables**			
LHD governance type	2019 LHD governance classification	- unit of state government - unit of local government - unit governed by both state and local authorities	- State - Local - Shared
LHD’s size of population served	3-level population categories	<50,000 50,000–499,999 ≥500,000	- Small - Medium - Large

Abbreviations: LHD, Local Health Department; PHAB, Public Health Accreditation Board; LBOH, Local Board of Health; NALBOH, National Association of Local Boards of Health; AIAN, American Indian or Alaska Native; NH, Native Hawaiian; PI, Pacific Islander; FTE, Full-Time Equivale.
